# The Regulatory Mechanisms and Control Technologies of Chilling Injury and Fungal Diseases of Postharvest Loquat Fruit

**DOI:** 10.3390/plants11243472

**Published:** 2022-12-12

**Authors:** Shen Zhang, Huimin Sun, Jingyi Wang, Junnan Shen, Fan He, Dongxiao Chen, Ying Wang

**Affiliations:** College of Ocean Food and Biological Engineering, Jimei University, Xiamen 361021, China

**Keywords:** loquat fruit, cold storage, chilling injury, lignification, fungal disease, postharvest treatment, regulatory mechanism

## Abstract

Loquat is a popular fruit widely cultivated all over the world. It is rich in minerals and carotenoids and has high commercial value. At room temperature, loquat fruit is impressionable to water and nutritional losses, physical damage, and microbial decay, resulting in a short postharvest life. Low-temperature storage is routinely used to prolong the shelf life of loquat fruit; however, cold storage can also lead to lignification of flesh tissue, which is one of the major symptoms of chilling injury (CI), reducing the quality and economic value of the fruit. In addition, fruit decay caused by microbial infection is another important reason for postharvest losses of loquat. To reduce quality deterioration and optimize the postharvest storage strategies of loquat fruit, considerable progress has been made in the physiological and molecular biological studies of CI, microbial decay, and preservation technologies of loquat fruit during the postharvest phase in recent decades. This review summarizes the current research progress and provides a reference for the improvement of loquat fruit quality.

## 1. Introduction

Loquat (*Eriobotrya japonica* Lindl.) is a subtropical evergreen fruit tree that originated in southeast China and belongs to the Maloideae subfamily of Rosaceae [1]. Nowadays, loquat is cultivated in more than 30 countries around the world, including Asia (China, Japan, South Korea, India, and Pakistan), Mediterranean (France, Italy, Greece, Spain, and Turkey), and American countries (Brazil, Chile, Argentina, and United States) [2,3,4]. Loquat in China is mainly distributed in provinces south of the Yangtze River, with the cultivation area and total output ranking first in the world. Loquat blossoms in autumn or early winter and matures in spring to early summer. The fruit is soft, juicy, and nutritious, containing a large number of carbohydrates, carotene, vitamins, and a variety of minerals and other nutrients. According to the “Compendium of Materia Medica”, loquat also has medicinal value for preventing diabetes, improving the immune system, regulating blood pressure, and relieving cough and asthma [5]. Therefore, loquat fruit is known as healthy fruit and is favored by consumers.

Loquat fruit is susceptible to nutrient loss, mechanical damage, and microbial decay, leading to a short postharvest storage life of approximately 10 days when stored at room temperature [3,6]. Cold storage is the primary method for loquat fruit preservation, which can effectively control the nutrient loss, senescence, and postharvest decay, thereby prolonging the fresh eating period and shelf life of loquat. However, refrigeration can also lead to chilling injury (CI), which exhibits a unique phenomenon in accelerating lignin accumulation and increasing fruit firmness, resulting in a leathery texture, and ultimately reducing consumer acceptance [7,8]. In addition, fruit decay caused by pathogen infection during postharvest storage and handling is an urgent problem to be solved in the global food supply chain, and loquat is no exception. In this review, we focus on the CI and microbial infection that are the main causes for quality deterioration and postharvest decay of loquat fruit and summarized the recent great progress on the mechanisms of postharvest CI and diseases, and preservation technologies of loquat fruit, providing a reference for the future research and development of preservation and disease prevention technology.

## 2. CI of Loquat Fruit after Harvest

### 2.1. Key Factors Causing CI of Postharvest Loquat Fruit

Plant cells respond to low temperature by regulating physiological metabolism in the short term. Otherwise, it is manifested as CI due to physiological damage [9]. Loquat fruit usually suffer from CI when stored below 5 °C and exhibit severe symptoms of lignification, internal browning, and loss of juiciness when stored at 0 °C [10]. Depending on the color of the fruit, loquats are divided into red and white. The content of carotenoids in red-fleshed loquat is higher, while white-fleshed loquat is higher in glutamate [11]. Studies have shown that red-fleshed loquat will suffer from CI during postharvest cold storage, resulting in lignification, while white-fleshed loquat will not [12,13].

The cell wall is a physical barrier against abiotic stress, and previous studies have suggested that the development of CI in loquat fruit is owed to abnormal changes in the cell wall, giving rise to cellular injury and facilitating the postharvest loquat fruit decay [14,15]. The primary cell wall is mainly composed of proteins and polysaccharides such as cellulose, hemicellulose, and pectin. Hemicellulose mainly includes xylan, xyloglucan, glucomannan, and mannan, which is a polysaccharide other than cellulose or pectin. Xylan is the principal component of hemicellulose in the secondary cell wall of dicotyledonous plants, and xyloglucan is the most abundant hemicellulose in the primary cell wall [16,17]. Studies have shown that the expression of xylan- and xyloglucan-related enzyme genes increase in refrigerated loquat fruit, leading to the accumulation of xylan and xyloglucan, thereby increasing the content of hemicellulose [18]. Secondary cell wall formation is accomplished by adding lignin, which is a complex polyphenolic polymer derived from phenylalanine, to the polysaccharide network composed of cellulose and hemicellulose. Lignin provides important biological functions for cell walls, such as structural support, water tightness, and resistance to environment stimuli [14]. The changes of cell wall polysaccharides in loquat fruit during softening are contrary to the typical depolymerization process, which shows that the contents of water-soluble pectin and cyclohexane diamine tetraacetic acid (CDTA)-soluble pectin decrease, and the contents of Na_2_CO_3_-soluble pectin, hemicellulose, and cellulose increase. Homogalacturonic acid (HG) is the most abundant cell wall pectin with a 70–80% methyl esterified form [19]. Pectin methylesterase (PME) catalyzes the removal of methyl esters from the (1-4) α-d-GalA backbone of HG and enhances the sensitivity of polygalacturonase (PG) and pectin lyase (PL) to HG hydrolysis [17,18]. The PG activity increases and the insoluble pectin content decreases in loquat fruit after 1-Methylcyclopropylene (1-MCP) treatment, thus alleviating the CI during cold storage [14]. Transcriptome analysis of loquat fruit in response to cold storage shows that the coding genes of α-1,4-galacturonic acid transferase and PME are downregulated first and then upregulated. However, the PME inhibitor (PMEI) coding gene is upregulated sharply in the early stage and maintains at a high level. Therefore, the activity of PME in loquat fruit stored at low temperature is significantly inhibited, which affects the removal of pectin methyl ester in HG. In addition, the expression level of endo-/exo PG and PL coding genes decrease remarkably after cold storage, which inhibits the degradation of pectate [18].

The cell membrane, which is considered to be the primary location of CI, undergoes a phase transition under low-temperature stress and gradually changes from liquid crystal to a solid state, which has a negative effect to the structure and function of the membrane [9,20]. The structural and functional changes are the main responses to low-temperature stress, followed by the increase in cell membrane permeability and damage [21,22]. Besides, lipids are an important component of cell membranes, and phospholipids are the main structural components of cell membranes, accounting for the majority of lipids. Studies have shown that membrane lipid peroxidation and decomposition are the main factors leading to membrane damage [9,20]. Three enzymes, phospholipase D (PLD), lipase, and lipoxygenase (LOX), have been reported as vital enzymes in cell membrane lipid metabolism that are involved in chilling tolerance enhancement [23]. Among them, PLD specifically hydrolyzes structural membrane phospholipids into phosphatidic acid (PA) and hydrolysate. Under the action of lipase, PA is then hydrolyzed into free fatty acids, and the hydrolysate plays a role in signal transduction as a second messenger [24,25,26]. Membrane lipid peroxidation, reflected in decreased unsaturation of membrane phospholipids and fatty acids and increased malondialdehyde (MDA) levels, is another cause of membrane damage. LOX destroys the bilayer of phospholipid by oxidizing the carbon–carbon double bonds of unsaturated fatty acids, such as linoleic acid and linolenic acid, thereby reducing the fluidity of cell membranes [27]. It has been demonstrated that 2,4-epibrassinolide (EBR) maintains high levels of unsaturated fatty acids by reducing LOX and PLD activities to retard the reduction of oleic, linoleic, and linolenic acids, consequently enhancing cold tolerance of loquat fruit [28]. In addition, loquat treated with chitosan/nano-silica or 1-MCP also has similar effects to alleviate the CI of loquat fruit [29,30].

**Figure 1:**
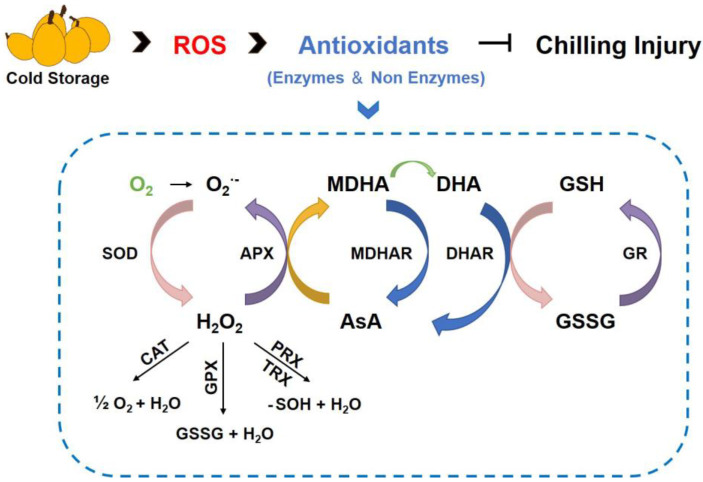
The antioxidant system involved in the control of ROS during cold storage of loquat. SOD, superoxide dismutase; CAT, catalase; APX, ascorbate peroxidase; GPX, glutathione peroxidase; PRX, peroxiredoxin; TRX, thioredoxin; MDHA, monodehydroascorbate reductase; MDHAR, dehydroascorbate reductase; DHA, dehydroascorbate; DHAR, dehydroascorbate reductase; GR, glutathione reductase; GSH, reduced glutathione; GSSG, oxidized glutathione; AsA, ascorbic acid.

Reactive oxygen species (ROS) play an important role in plant response to environmental stress, and oxidative damage is regarded as an early response of sensitive tissues to low temperature [31]. Normally, ROS production and elimination are in dynamic equilibrium, but low temperature can lead to excessive ROS, such as hydrogen peroxide (H_2_O_2_), superoxide anion (O_2_^•−^), and hydroxyl radicals (OH•), to cause oxidative stress [32,33]. ROS have high chemical activity and a relatively short half-life. Overproduction of ROS disrupts cellular homeostasis, causes impairments in DNA, proteins, and accelerates membrane lipid peroxidation, eventually leading to cell death [34,35,36]. To maintain ROS homeostasis and avoid oxidative damage, plants have evolved a complex antioxidant system consisting of both enzymatic and non-enzymatic antioxidant components. Enzymatic antioxidants contain superoxide dismutase (SOD), ascorbate peroxidase (APX), catalase (CAT), monodehydroascorbate reductase (MDHAR), dehydroascorbate reductase (DHAR), glutathione reductase (GR), etc., while non-enzymatic antioxidants include ascorbic acid (AsA), glutathione (GSH), carotenoids, and tocopherols, etc. [37]. These antioxidant enzymes, which are located in different parts of plant cells, work synergistically to detoxify ROS. Among them, SOD is the first line of defense to convert O_2_^•−^ into H_2_O_2_. CAT, APX, and GPX then scavenge H_2_O_2_. However, compared with CAT, APX requires an AsA and/or a GSH regenerating cycle, which consists mainly of GR, DHAR, and MDHAR. The AsA-GSH cycle is also crucial for ROS scavenging and preventing oxidative damage (Figure 1) [34,38]. Previous studies have indicated that increasing the activity of antioxidant enzymes (SOD, CAT, APX, GR, MDHAR, and DHAR) and enhancing the ASA-GSH cycle system are beneficial to alleviating oxidative damage and reducing the CI of loquat fruit [39,40,41,42]. Furthermore, Hou et al. [43] explored the relationship between ROS scavenging system and CI of loquat at a molecular level. The results indicated that CaCl_2_ treatment activated the expression of *EjAPX*, *EjGR*, *EjMDHAR*, and *EjDHAR* to eliminate ROS, finally alleviating the CI in loquat fruit.

### 2.2. Transcriptional Regulation of Lignification in Postharvest Loquat Fruit Induced by CI

Lignification is a common symptom of many low-temperature-sensitive fruit, such as mangosteen [44], pear [45], kiwifruit [46], and loquat [47], which is attributed to the accumulation of lignin in plant secondary cell walls. Under improper low-temperature conditions, lignification leads to the decline of fruit quality and seriously limits the storage life of fruit [48]. Lignin deposition comprises the synthesis and polymerization of monolignols, which are synthesized via the phenylpropanoid pathway. Under the catalysis of l-phenylalanine ammonia-lyase (PAL), phenylalanine is deaminated to form cinnamic acid. Cinnamic acid is then modified sequentially by a series of enzymes, such as cinnamate 4-hydroxylase (C4H), 4-coumarate: coenzyme A ligase (4CL), cinnamyl alcohol dehydrogenase (CAD), and peroxidase (POD), to catalyze the production of three monolignols called p-hydroxyphenyl (H), guaiacyl (G), and syringyl (S) lignin units, respectively [49,50,51]. These monomers are subsequently activated by peroxidase (PRX)/H_2_O_2_ and laccase (LAC)/O_2_ system located in the apoplast and then polymerized into lignin by oxidative coupling [52,53]. For the past few years, several structural genes involved in lignin biosynthesis have been identified, such as *EjPAL1* [12], *Ej4CL1/5* [50,54], *EjCAD5* [55], *EjCAD like* [56], and *EjCCoAOMT* [57]. Their transcript level increases under low temperature, which is positively correlated with the increase of fruit firmness and lignin content [58]. However, the precise regulatory mechanisms of these enzymes remain unclear.

**Figure 2:**
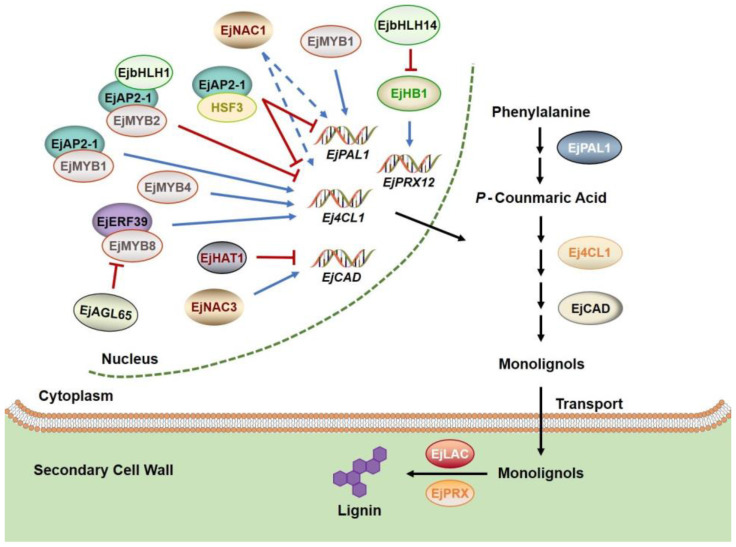
Current understanding of the transcriptional network of lignin biosynthesis in loquat. The blue arrow lines indicate activation, and the red blunted lines indicate repression. The dashed arrows represent indirect regulation. The overlapping ellipses represent protein–protein interactions.

In recent years, the molecular mechanism of loquat fruit lignification has been deeply studied at the transcriptional level (Figure 2). It has been demonstrated that multiple transcription factor families are involved in the regulation of lignin biosynthesis. EjMYB1 and EjMYB2 are the first transcription factors found to be involved in lignification of loquat fruit [59]. EjMYB1 can activate the expression of genes *EjPAL1*, *Ej4CL1*, and *Ej4CL5* related to lignin biosynthesis, and transient overexpression of *EjMYB1* in tobacco leaves triggers lignin accumulation. Another two *MYB* family genes, *EjMYB4* and *EjMYB8*, have also been shown to positively regulate lignin accumulation via directly binding to the AC-element of *Ej4CL1* promoter. Nevertheless, transactivation assays indicate that *EjMYB2* is a repressor of loquat fruit lignification [54,60]. Interestingly, EjODO1 which is an R2R3 type MYB transcription factor, also shows a high binding affinity to the *Ej4CL1* promoter. However, it appears to be a regulator of lignin biosynthesis in vegetative organs and early fruit development, rather than fruit maturation or postharvest lignification [61]. NAC-domain transcription factors are plant-specific transcriptional regulators that are considered to be ‘master switches’ for secondary cell wall formation because of their operation at the top layer of regulatory networks [62]. At present, there is no evidence for a physical interaction between NAC and structural genes. However, they have been shown to regulate a series of downstream transcription factors, such as MYB, which in turn regulate the biosynthetic genes for secondary cell wall deposition [63]. In loquat, EjNAC3 can directly bind to the promoter of *EjCAD-like* to regulate lignin biosynthesis induced by low temperature [56]. It has been found that EjNAC1 activates the promoter of *EjPAL1* and *Ej4CL1* to regulate lignin production. However, yeast one-hybrid experiments show that EjNAC1 can not directly bind to the promoter of *EjPAL1* and *Ej4CL1*. Therefore, the regulatory mechanism of EjNAC1 involved in fruit lignification is still unknown [64]. Furthermore, a MADS-box family transcription factor, EjAGL65 (belonging to the Mδ subgroup), has been verified to negatively regulate *EjMYB8* expression during cold-induced lignification [53].

It is well known that APETALA2/ETHYLENE RESPONSIVE FACTOR (AP2/ERF) transcription factors are of great importance for regulating plant response to low-temperature stress and lignin biosynthesis. EjAP2-1 indirectly regulates loquat fruit lignification by interacting with EjMYB1 or EjMYB2 [65]. In addition, EjERF39 is capable of recognizing the DRE element in the *Ej4CL1* promoter and transactivating its expression. Moreover, EjERF39 can also form a complex with EjMYB8 to synergistically activate the expression of *Ej4CL1*, thereby regulating low-temperature-induced lignification of loquat fruit [66]. Therefore, EjMYB8 is controlled at the protein level by EjERF39 and the mRNA level by EjAGL65. Zeng et al. [67] found that EjHSF transcription factor might participate in CI and lignification process of loquat fruit through two different pathways: EjHSF1 transcriptionally regulated *EjHsp* genes related to cold tolerance, while EjHSF3 directly bound to the promoter of lignin biosynthesis-associated genes or interacted with EjAP2-1 to regulate loquat lignification. Based on the RNA-seq, *EjbHLH1* was cloned from loquat fruit. Further analysis indicated that EjbHLH1 formed a ternary complex with EjMYB2 and EjAP2-1 to repress the expression of *Ej4CL1*, thereby inhibiting the biosynthesis of lignin [55]. To date, a new regulatory complex EjbHLH14-EjHB1-EjPRX12 has been identified, revealing the molecular mechanism of methyl jasmonate (MeJA) alleviating low-temperature-induced lignification of loquat fruit. During postharvest storage, the expression of *EjPRX12* has a positive correlation with lignin accumulation, and EjHB1 is confirmed to be its upstream regulator. Overexpression of *EjPRX12* and *EjHB1* in *Arabidopsis* contributes to lignin accumulation. Moreover, EjbHLH14, a JA signaling pathway repressor, physically interacts with JASMONATE ZIM-DOMAIN (JAZ) to inhibit the activity of the *EjHB1* promoter, ultimately alleviating low-temperature-induced lignification [68]. In addition, a homeobox-leucine zipper protein, EjHAT1, has been demonstrated to regulate the lignification of loquat fruit in response to heat treatment by suppressing the activity of *EjCAD5* promoter [55]. Taken together, this direct evidence provides an opportunity to reveal the interaction mechanism between loquat fruit lignification-related transcription factors, and to elucidate the regulatory mechanisms of downstream structural genes, such as enzymes and receptor proteins involved in lignification at the transcriptional level.

## 3. Postharvest Diseases of Loquat Fruit

Fruit decay caused by microbial invasion is another major reason for postharvest loss of loquat fruit. Among them, fungal disease is the main type of loquat disease (Table 1). A variety of fungi have been confirmed to infect loquat, including *Alternaria alternata*, *Fusarium solani*, *Colletotrichum acutatum,* and others [69,70,71,72]. Anthracnose rot caused by *C. acutatum* or *Colletotrichum gloeosporioides* is the main fungal disease of postharvest loquat fruit [73,74,75,76]. The most severe stage of anthracnose begins at the seedling and fruit ripening stage, especially when the weather is high in humidity and continuous rainfall [77]. According to the biological characteristic analysis of *C. acutatum*, the optimum growth temperature of the mycelium is 26–28 °C, and the optimum pH is 6. When the relative humidity reaches 100%, the spore germination rate reaches the highest level, but when the relative humidity is below 90%, the spores do not germinate. Further analysis shows that the lethal temperature of the spores is 50 °C for 5 min or 55 °C for 2 min [77].

Apart from anthracnose, grey rot caused by *Pestalotiopsis eriobotryfolia* and black rot caused by *Alternaria tenuis* are also common in harvested loquat fruit, but their infection rates are lower than that of anthracnose [78,79]. The strain isolated from rotting loquat fruit in Spain was identified as *Neopestalotiopsis clavispora* by molecular phylogenetic analysis [80]. Moreover, fungi with high sequence similarity have also been found in rotting loquats from Japan and Pakistan [81,82]. Additionally, the transmission of conidia of *Fusicladium eriobotryae* was detected in two loquat orchards in Spain [83]. *Geotrichum candidum* is a worldwide fungus belonging to the *Ascomycota*, which can cause sour rot in ripe and over-ripe fruit such as peaches and nectarines. The disease symptoms include a brown, watering, soft corrosion, and a thin layer of white mycelium growing on the fruit surface [84,85]. This fungus is highly tolerant to pH (5–7) and temperature (5–38 °C) and is therefore a common pathogen of various fruit [86]. Hafeez et al. [87] found that *G. candidum* from Pakistan could also cause postharvest sour rot in loquat fruit. Furthermore, according to the statistics, ‘Algerie’ is currently the main loquat cultivar in Spain and its most common disease is caused by *A. alternata*, followed by *Penicillium expansum*. In addition, *Botrytis cinerea*-induced decay is observed in a large number of injured and refrigerated fruit, while anthracnose is frequently found in fruit after surface disinfection. The incidence rate of loquat fruit caused by other pathogens, such as *Pestalotiopsis clavispora* and *Diplodia seriata,* is low [75]. Currently, *Rhizopus stolonifer* has been identified from rotting loquat fruit in Pakistan [88]. In short, the fungi that cause postharvest decay of loquat are diverse and seriously affect the yield of loquat and cause huge economic losses worldwide every year. However, the current studies mainly focus on the isolation and identification of pathogens, and there are few reports on the response mechanisms and the interaction between pathogens and loquat fruit.

## 4. Postharvest Technologies of Loquat Fruit

**Figure 3:**
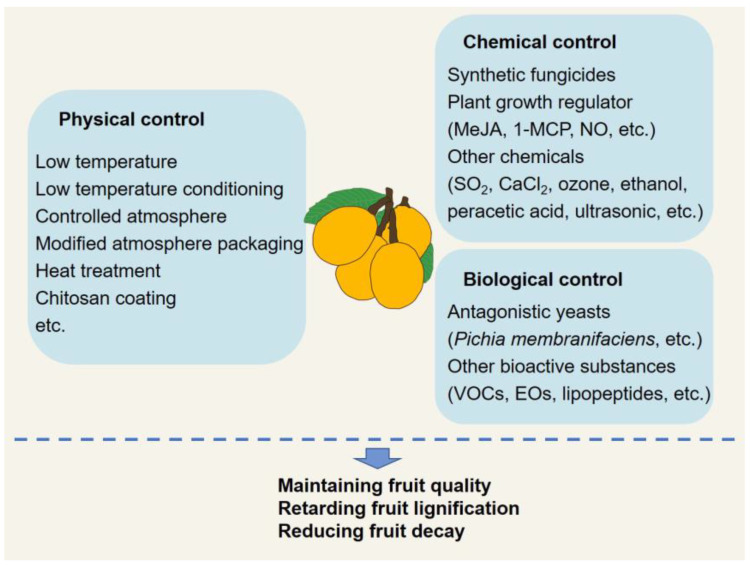
Postharvest technologies of loquat fruit.

Since loquat fruit is perishable and has a short postharvest life, various strategies, such as physical, chemical, and biological technologies, have been employed to maintain the quality of loquat fruit after harvest and reduce economic losses (Figure 3).

### 4.1. Physical Technologies

Low temperature can effectively reduce the fruit respiration rate, regulate the reaction of enzymes, and inhibit the growth of pathogenic microorganisms, ultimately ensuring the storage quality of fruit after harvest [89,90]. Hence, low-temperature storage becomes a widely used method to reduce fruit decay, maintain quality, and prolong the shelf life of loquat. The optimum storage temperature of loquat fruit is related to the susceptibility of cultivars to CI, and the minimum safe temperature range to avoid CI is 0–10 °C [3]. Studies have shown that ‘Jiefangzhong’ is suitable for storage at 6–8 °C, ‘Zhaozhong’ is suitable for storage at 8–10 °C, ‘Wuxing’ can be stored at 1 °C for 30 days, and ‘Mogi’ loquat can be maintained for up to 30 days at 1 or 5 °C [3,91,92]. It has been reported that the ability of loquat to scavenge and reduce DPPH radicals decreases when stored at 20 °C, while the low-temperature storage maintains high DPPH free radical scavenging ability and reducing capacity [93]. However, CI caused by low-temperature storage below 5 °C is the main limitation of long-term storage of loquat [3], whereas low-temperature conditioning (LTC) is effective in reducing fruit CI. Jin et al. [94] demonstrated that LTC treatment (10 °C for 6 days followed by 1 °C for up to 5 weeks) might enhance the chilling tolerance of loquat fruit through increasing the endogenous glycine betaine content, which stabilized membranes under environmental stress, thus improving the energy state and reducing ion leakage and malondialdehyde content.

The combination of controlled atmosphere (CA) and cold storage is an ideal approach to extending the shelf life of loquat fruit. The respiration rate and the growth of microbes and insects can be easily controlled by lowering the oxygen concentration in the storage chamber and increasing the carbon dioxide concentration. Studies have shown that loquat fruit can be preserved for more than 50 days at 1 °C with normal fruit flavor and low decay incidence in CA with 10% O_2_ + 1% CO_2_. Besides, treatment with 70% oxygen for 24 h followed by storage in this CA has little effect on fruit flavor but stimulates ethanol accumulation in loquat fruit and reduces the activities of endo-PG, exo-PG, and polyphenol oxidase (PPO) [91]. High oxygen (90%) treatment effectively inhibits loquat respiration rate and PPO activity, and the flavor of the fruit is better than the control after storage for 35 days [95]. In addition, the weight loss and organic acid residues of loquat can be reduced by packaging them in a modified atmosphere (MA, 4 kPa O_2_ and 5 kPa CO_2_, 20 μm thick polyethylene film) at 5 °C. The storage period of the fruit under this MA can reach 2 months and the fruit quality is better than control [96]. Similarly, using modified atmosphere packaging (MAP) (2–4 kPa CO_2_ and 16–18 kPa O_2_; micro-propylene) prevents the weight loss of fruit, delays color change and softening, and reduces the content of organic acid and sugar [97]. Compared with the MAP storage (polyethylene bag of 0.01 mm thickness at 1 and 6 °C), CA storage (10% O_2_ + 1% CO_2_) controls fruit decay more effectively due to the reduced PPO activity and oxidative stress [91,98]. Furthermore, the decay incidence of a white-flesh loquat cultivar ‘Qingzhong’ that packaged in nano-SiO_2_ bags is 42.84% lower than control after 12 d. Meanwhile, nano-SiO_2_ packing maintains higher contents of total soluble solids, titratable acid, and total phenolic and soluble sugar in loquat fruit, which is conducive to better quality and extended shelf life [99].

There are many other physical technologies applied in postharvest loquat fruit that have been reported. For instance, heat treatment (HT, 38 °C for 5 h, then store at 1 °C) reduces the internal browning in refrigerated ‘Jiefangzhong’ loquat fruit, which may be attributed to the preservation of membrane integrity and the higher ratio of unsaturated/saturated fatty acid [100]. Moreover, loquat fruit treated with HT (40 °C for 4 h followed by transfer to 0 °C) shows alleviated lignification. Transcriptome analysis indicates that biological processes, such as stress responses, cell wall and lignin metabolism, hormone metabolism, and metal ion transport, are remarkably affected under HT treatment [58]. Besides, the activities of PAL, C4H, and 4CL are positively correlated with loquat fruit lignification, and the suppression of these activities by HT (40 °C for 4 h followed by transfer to 0 °C) markedly reduces the lignification of loquat fruit [50]. Accumulated evidence suggests that hot water (HW) treatment is effective in reducing CI in papaya [101], tomato [102], and mango fruit [103]. Zhang et al. [104] found that HW dipping (45 °C for 10 min) treatment significantly enhanced the chilling tolerance of loquat fruit. Interestingly, the combined treatment of HW and glycine betaine (GB, 10 mM GB at 45 °C for 10 min) was more effective in reducing CI and maintaining quality parameters than HW or GB alone. The effect was due to prevention of oxidative damage and promotion of the accumulation of endogenous proline and γ-aminobutyric acid contents. In addition, chitosan coating followed by cold storage (7 °C) maintains the quality and remarkably prolong the shelf life of loquat fruit, which reduces flesh browning, maintains fruit firmness, and minimizes the losses of total polyphenol, carotenoid, and ascorbic acid. The nutraceutical value of loquat fruit can be enhanced up to 21 days by chitosan coating with cold storage [105,106].

### 4.2. Chemical Technologies

1-MCP, an ethylene inhibitor, has been widely applied to extend the shelf life of various climacteric and non-climacteric fruit, which can effectively delay fruit ripening and softening and improve disease resistance by competitively binding ethylene receptors [107,108,109,110]. It has been demonstrated that 1-MCP treatment modifies the fatty acid and cell wall polysaccharide composition, as well as enhances antioxidant enzyme activity to reduce CI effectively in postharvest loquat fruit [111,112]. Further study indicates that 1-MCP may alleviate loquat CI by regulating the transcription of *EjETR1,* which is located in the endoplasmic reticulum membrane to perceive ethylene [113,114]. Additionally, Cao et al. [115] revealed that 1-MCP treatment markedly increased the activities of two defense-related enzymes, chitinase and β-1, 3-glucanase, thus effectively controlling the anthracnose rot of loquat fruit caused by *C. acutatum* infection. MeJA is a natural plant signaling molecule that is involved in various physiological processes such as fruit ripening and responses to environmental stress [116]. It has been reported that postharvest application of MeJA has a positive effect on the overall quality of loquat fruit, which is attributed to that MeJA increases the phenols contents in loquat fruit by inhibiting PPO activity, thus enhancing the antioxidant activity of loquat fruit [111]. Moreover, MeJA combined with hot air treatment can activate the activities of antioxidant enzymes and inhibit lignin biosynthesis, thereby reducing the CI of loquat fruit [117]. MeJA also plays a vital role in defense response. On the one hand, MeJA treatment directly inhibits spore germination, germ tube elongation, and mycelial growth of *C. acutatum*. On the other hand, treatment with 10 μmol/L MeJA remarkably enhances H_2_O_2_ generation in loquat fruit, which may be a signal to induce disease resistance against *C. acutatum* infection [118]. 

Sulfur dioxide (SO_2_) treatment significantly inhibits the browning of the internal tissues and reduces the incidence of fruit decay, finally prolonging the storage life of loquat [119]. Further analysis shows that SO_2_ application maintains the balance between the generation and detoxification of ROS to inhibit ROS accumulation, thereby preventing the flesh lignification of loquat fruit [120]. Additionally, l-cysteine, a compound of sulfhydryl, is used as an alternative to SO_2_ to effectively inhibit loquat juice browning. The modes of action of thiol may be attributed to the formation of a thiol-conjugated reaction product [121]. Babu et al. [122] found that soaking loquat fruit in 3% CaCl_2_ retained maximum total soluble solid and also delayed weight loss for 24 days compared with 1% and 2% CaCl_2_. Besides, CaCl_2_ treatment combined with low-temperature storage was effective in retaining fruit firmness and preventing browning, which mainly depended on maintaining the stability of the membrane and increasing cell wall strength [123]. Recently, researchers found that the alleviation effect of CaCl_2_ treatment on loquat fruit CI was associated with the modulation of ROS homeostasis, which enhanced the activities of antioxidant enzymes and AsA-GSH cycle system to quench over-accumulated ROS [43]. 

Nitric oxide (NO) is a ubiquitous second messenger in plants, which plays a crucial role in alleviating postharvest stresses [124]. NO treatment can reduce ethylene production, respiratory rate, and lipid oxidation, as well as improve antioxidant enzyme activity and increase endogenous NO, which contribute to maintaining cell integrity and avoiding low-temperature damage. Moreover, endogenous NO has the potential in regulating plant defense responses, which can counteract ROS and reduce CI [125]. The effects of endogenous NO on loquat fruit have been investigated and the results show that low temperature at 1℃ causes a significant increase in endogenous NO level, which triggers the activity of antioxidant enzymes to scavenge ROS and reduce lipid peroxidation and cell membrane damage, thereby enhancing the tolerance of fruit to low-temperature stress [126]. In addition, the application of suitable exogenous NO on young loquat fruit accelerates the mitochondria AsA-GSH circulation metabolism, thus reducing the oxidative damage of fruit during cold storage and improving the cold resistance capacity of loquat [127]. Interestingly, there is an interaction between Ca^2+^ and NO signal transduction in loquat seedlings in response to chilling stress. Co-treatment with CaCl_2_ and sodium nitroprusside (SNP, an exogenous NO donor) has a synergistic effect, resulting in the decrease of intracellular H_2_O_2_ and MDA accumulation under low-temperature stress [128].

Salicylic acid (SA), a natural and safe compound, has been found to generate a wide range of metabolic and physiological responses in plants and contributes to the fruit defense systems [129]. In loquat, 5 mM SA treatment effectively reduces fruit decay by controlling cell membrane electrolyte leakage, decreasing respiration and ethylene production, maintaining flesh firmness, and increasing antioxidant enzymes activities [130].

It has been found that loquat fruit treated with 150 mg/m^3^ ozone effectively delays the increase of lignin content and cell membrane permeability and reduces the fruit decay rate [131]. Furthermore, ethanol is able to inhibit the postharvest anthracnose of loquat fruit, and its modes of action may be attributed to directly inhibiting the growth of pathogen growth or indirectly inducing fruit disease resistance [132]. Currently, Ling et al. [133] demonstrated that the combination of peracetic acid and ultrasonic treatment effectively maintained the fruit quality and significantly reduced fruit decay of loquat during room temperature storage. In a word, chemical treatment has a significant effect on prolonging the shelf life of loquat fruit, but chemical treatment has a concentration effect, which is related to variety, fruit maturity, treatment time, and storage temperature. Besides, the use of serine protease (120 mg/L) effectively reduces the postharvest decay caused by *C. acutatumis* of loquat fruit. Its modes of action are related to inhibiting the germination and growth of *C. acutatum* and delaying the accumulation of ROS through improving the scavenging ability of both enzymatic and non-enzymatic antioxidant materials [134].

### 4.3. Biological Technologies

In the past decades, biological technology has been widely studied and is regarded as a safe and effective alternative to chemical technology in postharvest field. Several antagonistic yeasts have been reported to effectively inhibit postharvest decay of loquat fruit, including *Pichia membranifaciens*, *Pichia guilliermondii,* and *Metschnikowia pulcherrima* E1, which compete with pathogenic fungi for nutrition and space [74,135,136]. Furthermore, combined treatment with MeJA could markedly enhance the biocontrol activity of *P. membranifaciens* against anthracnose rot of loquat fruit, and its modes of action may be related to inhibiting the pathogen growth and enhancing the disease resistance of loquat fruit [112]. Liu et al. [137] found that hot air treatment in combination with *P. guilliermondii* was more effective in controlling anthracnose rot caused by *C. acutatum* compared with heat treatment or the use of *P. guilliermondii* alone. In addition to antagonistic yeast, a rhizobacterium, *Bacillus cereus* AR156, is considered to be effective in inducing disease resistance in loquat fruit against *C. acutatum* infection. The mechanisms of action are related to the initiation of H_2_O_2_ production and the expression of defense-related genes [138]. Similarly, *Bacillus amyloliquefaciens* HG01 can also effectively inhibit anthracnose rot in postharvest loquat fruit by directly inhibiting the growth of *C. acutatum*. Moreover, HG01 treatment ensures the good quality of loquat maintaining higher contents of total phenolic, amino acid, organic acid, and sugar compared with the control [139].

In recent years, the application of antifungal active substances produced by microorganisms has received extensive attention for fruit preservation after harvest. Volatile organic compounds (VOCs) generated by microorganisms, such as *Bacillus* sp., *Pseudomonas* sp., and *Streptomyces* sp., have suppressive effects on the growth and pathogenicity of fungi [140,141,142,143]. Indeed, VOCs released by *Bacillus methylotrophicus* BCN2 and *Bacillus thuringiensis* BCN10 can effectively inhibit the mycelial growth of five pathogenic fungi of loquat and reduce the disease incidence as well as lesion diameter of loquat fruit [144]. According to the results reported by Yan et al. [145], lipopeptide antibiotic iturin A produced by *B. amyloliquefaciens* MG3 inhibits the mycelia growth of *C. gloeosporioides* by increasing the permeability of cell membrane and MDA content and then controls the anthracnose rot of loquat. Additionally, botanicals have become the most innovative approaches being used in biological preservation, such as plant essential oils (EOs), which are classified by the Food and Drug Administration (FDA) as safe for use in food [146,147]. EOs of peppermint, cinnamon, fennel, and citronella have antifungal activity against phytopathogenic fungi, such as *Alternaria alternaria*, *Fusarium tabacinum*, *B. cinerea*, and *Aspergillus fumigates*, which cause serious diseases of postharvest fruit and vegetables [147,148,149,150]. Eight EOs isolated from the leaves of eucalyptus, thyme, lemongrass, moringa, tea tree, peel of lemon, pomegranate, and rhizome of ginger are all capable of inhibiting the mycelial growth of *A. alternata*. Among them, thyme oil has the best control effect on *A. alternata*-induced loquat fruit decay in a dose-dependent manner [151].

To sum up, a variety of physical, chemical, and biological methods can effectively control the CI and fungal diseases of loquat fruit after harvest, showing a broad commercial application prospect. However, many studies still focus on the detection of physiological and biochemical indexes of loquat fruit, and the regulatory mechanisms of these methods have not been thoroughly studied. In order to solve this problem for a long time and promote the application of related methods, it is necessary to use existing molecular genetic technologies to explore those in-depth regulatory mechanisms.

## 5. Conclusions

Loquat is rich in sugars, organic acids, phenolic substances, flavonoids, and vitamins, which are favored by consumers. However, CI caused by low-temperature storage and fruit rot caused by pathogen infection are the major problems of loquat preservation after harvest, which seriously affect its taste and commercial value. With the identification of various transcription factors that directly or indirectly regulate lignification, we have a preliminary understanding of the transcriptional regulation of lignification caused by CI. However, it is not enough to elucidate the transcriptional regulatory network and the interaction mode between transcription factors in response to CI in loquat. Although considerable progress has been made in the types of postharvest fungal diseases of loquat fruit, the biological characteristics of pathogens, the occurring rules of major diseases, and the preliminary control measures in recent years, there are few studies on the mechanism of loquat response to diseases. Future research should continue to explore loquat lignification-related transcription factors, determine their downstream targets and mechanisms of action, explore the molecular mechanisms of the interaction between loquat and pathogens, and then build a comprehensive loquat transcriptional regulatory network in response to CI and postharvest disease, thereby providing a theoretical basis for the development of safe and efficient postharvest preservation technologies of loquat.

## Figures and Tables

**Table 1:** List of fungi that cause postharvest decay of loquat fruit.

NO.	Fungal Pathogen	Authors and References
1	*Colletotrichum acutatum*	Liu, A.Y. [56]; Cao, S.F. [74]; Tziros, G.T. [70]; Abbas, M.F. [72]; etc.
2	*Colletotrichum gloeosporioides*	Palou, L. [75]; etc.
3	*Geotrichum candidum*	Michailides, T.J. [84]; Pitt, J.I. [85]; Hafeez, R. [87]; etc.
4	*Colletotrichum godetiae*	Juárez-Vázquez, S.B. [71]
5	*Alternaria tenuis*	Gu, H. [79]
6	*Alternaria alternata*	Tziros, G.T. [70]
7	*Pestalotiopsis eriobotryfolia*	Cai, P. [78]
8	*Fusarium solani*	Wu, D. [73]
9	*Neopestalotiopsis clavispora*	Palou, L. [80]
10	*Pestalotiopsis sensu*	Nozawa, S. [81]
11	*Neopestalotiopsis clavispora*	Abbas, M.F. [82]
12	*Fusicladium eriobotryae*	González-Domínguez, E. [83]
13	*Penicillium expansum*	Palou, L. [75]
14	*Botrytis cinerea*	Palou, L. [75]
15	*Pestalotiopsis clavispora*	Palou, L. [75]
16	*Diplodia seriata*	Palou, L. [75]
17	*Rhizopus stolonifer*	Aslam, M.F. [88]

## Data Availability

Not applicable.

## Abbreviations

AsA: ascorbic acid; APX: ascorbate peroxidase; CA: controlled atmosphere; CAD: cinnamyl alcohol dehydrogenase; CAT: catalase; CDTA: cyclohexane diamine tetraacetic acid; C4H: cinnamate 4-hydroxylase; CI: chilling injury; DHAR: dehydroascorbate reductase; DPPH: 1,1-Diphenyl-2-picrylhydrazyl radical 2,2-Diphenyl-1-(2,4,6-trinitrophenyl) hydrazyl; EBR: 2,4-epibrassinolide; EOs: essential oils; FDA: Food and Drug Administration. GR: glutathione reductase; GSH: glutathione; HG: homogalacturonic acid; LAC: laccase; LOX: lipoxygenase; MDA: malondialdehyde; LTC: low-temperature conditioning; MA: modified atmosphere; MAP: modified atmosphere packaging; 1-MCP: 1-methylcyclopropylene; MeJA: methyl jasmonate; MDHAR: monodehydroascorbate reductase; NO: nitric oxide; PA: phosphatidic acid; PAL: l-phenylalanine ammonia-lyase; PG: polygalacturonase; PL: pectin lyase; PLD: phospholipase D; PME: pectin methylesterase; PMEI: PME inhibitor; PPO: polyphenol oxidase; SOD: superoxide dismutase; 4-CL: 4-coumarate: coenzyme A ligase; POD: peroxidase; PRX: peroxidase; ROS: reactive oxygen species; SNP: sodium nitroprusside; SO_2_: sulfur dioxide; VOCs: volatile organic compounds.
